# Computed Tomography Images of a Benign Case of Gastric Pneumatosis in a Returning Traveler

**DOI:** 10.7759/cureus.68916

**Published:** 2024-09-08

**Authors:** Matthias Barden, Chris D Kim, Muffadel A Taher

**Affiliations:** 1 Emergency Medicine, Eisenhower Health, Rancho Mirage, USA; 2 Radiology, Eisenhower Health, Rancho Mirage, USA

**Keywords:** computed tomography abdomen, gastric pneumatosis, gastroenteritis complication, travel-related infection, vigorous vomiting

## Abstract

A 70-year-old male presented to the emergency department with travel-associated vomiting, diarrhea, and abdominal pain. He was found to have gastric pneumatosis on computed tomography. His serum lactic acid level was within normal limits, and he had a benign clinical course.

Gastric pneumatosis can be found in a wide spectrum of clinical situations, from benign to life-threatening. Causes can include ischemia, infections with gas-producing organisms (emphysematous gastritis), or various situations that result in increased intraluminal pressure. As this patient had not recently undergone any endoscopic procedures and had a benign presentation and clinical course, the cause, in this case, is presumed to be related to vigorous retching during a bout of traveler’s gastroenteritis.

## Introduction

We present a report on a benign case of gastric pneumatosis with associated computed tomography (CT) images. Emergency physicians will likely associate gastrointestinal tract pneumatosis with grave conditions such as ischemia or infection [1,2]. However, this CT finding is not specific to a particular disease process and may also be present in more benign settings [3]. The focus of this presentation is to highlight the CT image findings in gastric pneumatosis and to demonstrate that this finding may occur in benign cases.

## Case presentation

A 70-year-old male with a history of hypertension, chronic obstructive pulmonary disease, and bipolar affective disorder presented to the emergency department with chief complaints of nausea, vomiting, diarrhea, abdominal pain, and generalized weakness. He had recently returned to the United States after traveling to Mexico. While abroad, he developed diarrhea, with reported five to six loose watery bowel movements per day over the preceding week. A few days before his presentation, he had severe retching with dry heaves and some vomiting. He had not been able to tolerate oral intake since then and had some further retching and dry heaves on the day of presentation to the emergency department (ED).

**Figure 1:**
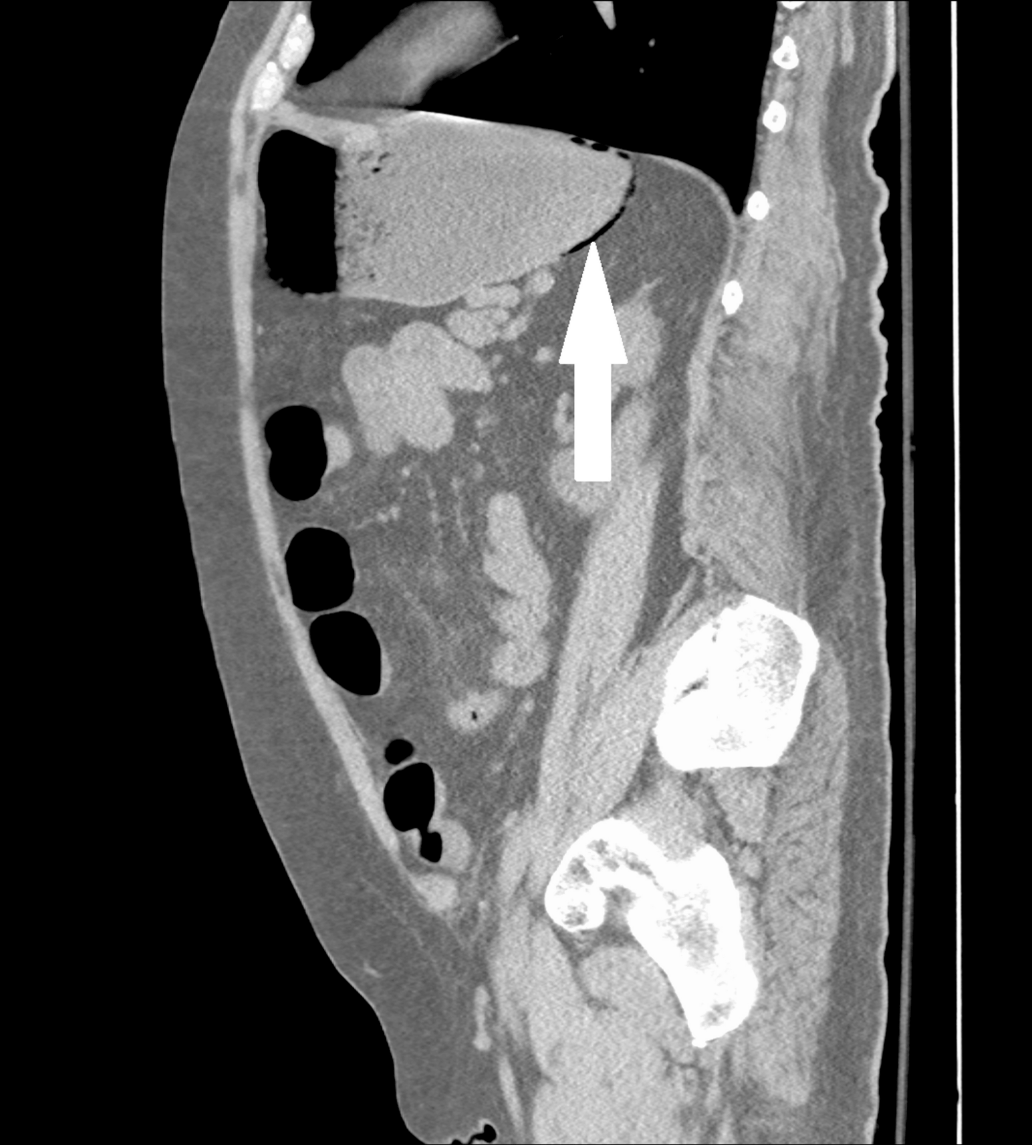
Computed tomography scan, sagittal view The normal gastric bubble of air is shown in the expected anti-dependent position, but there is an additional curvilinear tract of air (white arrows) along the posterior wall of the stomach, raising concern for gastric pneumatosis.

**Figure 2:**
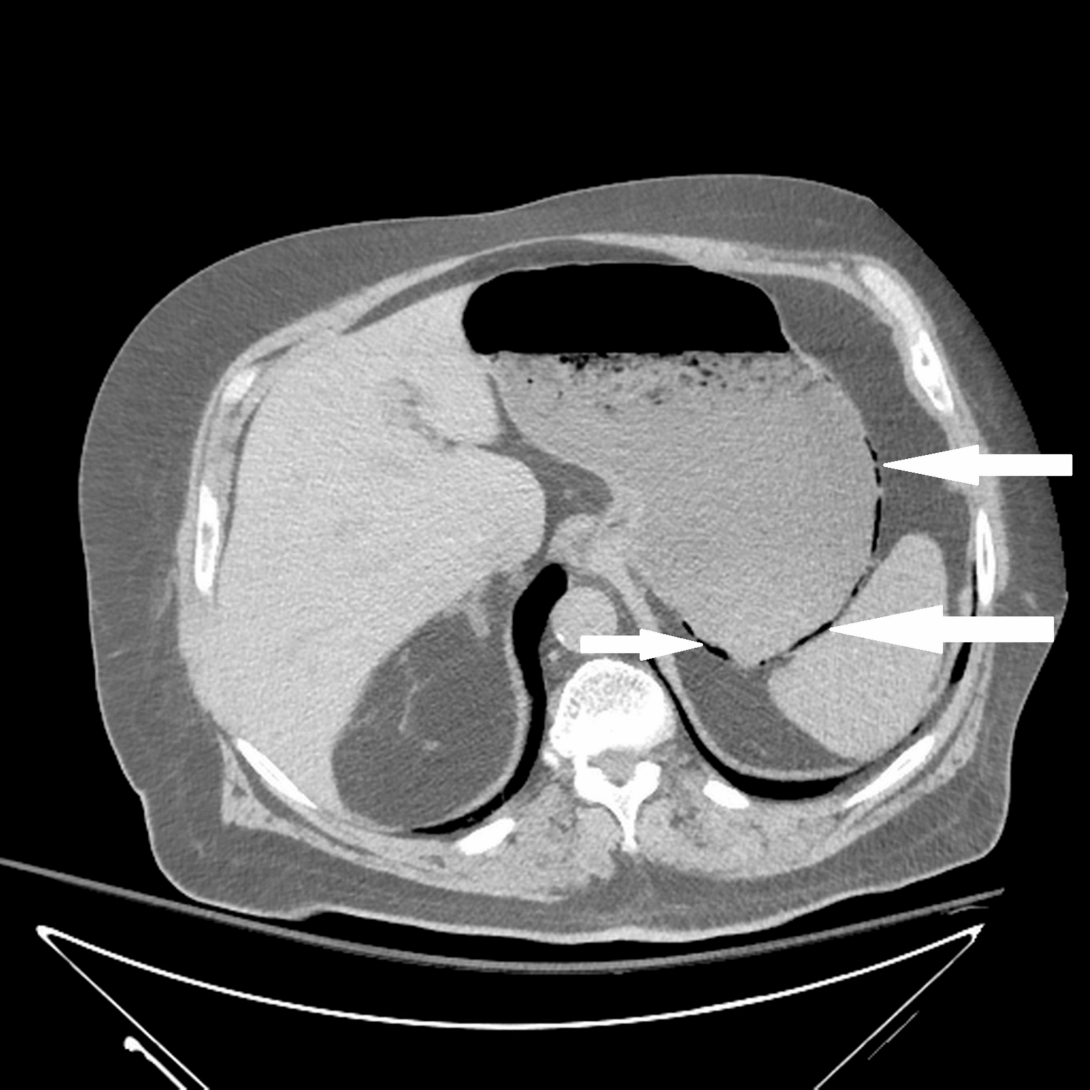
Computed tomography scan, axial view, showing curvilinear air (white arrows) tracking along the posterior wall of the stomach concerning for gastric pneumatosis

**Figure 3:**
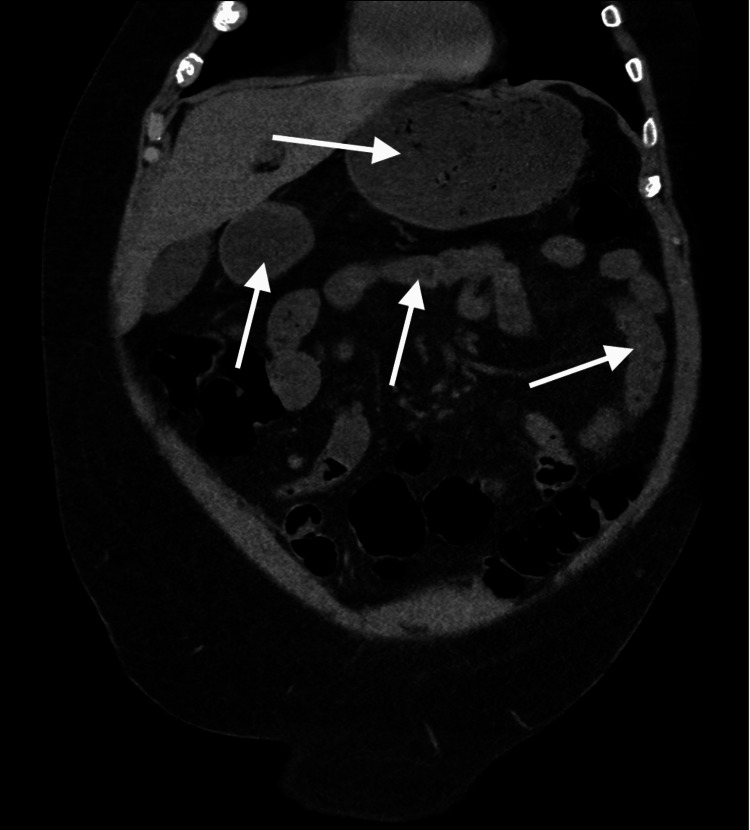
Computed tomography scan, coronal view, showing multiple loops of fluid-filled bowel (white arrows), consistent with the clinical picture of a diarrheal state, in this case presumed to be traveler’s acute gastroenteritis

In the ED, he had mild tachycardia and appeared clinically dehydrated but had an otherwise benign physical examination. His abdomen was soft, non-tender, and non-distended. As part of the workup for his abdominal pain and uncontrolled retching, he underwent laboratory testing and CT of the abdomen and pelvis. He was found to have gastric pneumatosis on CT (Figures 1, 2), as well as increased fluid content in the bowel, which correlates with his clinical picture of gastroenteritis (Figure 3).

A gastroenterologist was consulted, who recommended obtaining a serum lactic acid level to exclude ischemia, but noted that this was likely a rather incidental finding considering the generally benign clinical presentation.

Serum lactic acid level was measured at 1.0 millimole per liter (mmol/L) (reference range 0.5-2.2 mmol/L). The patient did have some mild decreased kidney function. He was admitted for intravenous fluids, monitoring, and repeat examination. He had a benign course and was discharged home the following day without incident.

## Discussion

Gastric pneumatosis is a finding that is not specific to one particular disease process [4]. It can be found in a wide spectrum of clinical situations, from benign to life-threatening such as ischemia, infections with gas-producing organisms (emphysematous gastritis), or increased intraluminal pressure [4,5]. While imaging is key in identifying the finding of gastric pneumatosis, it is unlikely to provide the underlying etiology or prognosis [6].

If gastric pneumatosis is caused by gastrointestinal ischemia, the serum lactic acid level is expected to be elevated [7]. Gastric pneumatosis secondary to infection with gas-producing organisms is termed emphysematous gastritis, which is rare and generally occurs in patients with severe systemic illness and predisposing factors [8].

Gastric pneumatosis can also be caused by increased intraluminal pressure, such as after an endoscopic procedure, due to gastric herniation or bowel obstruction, or after bouts of retching/vomiting [9,10].

As this patient had not recently undergone any endoscopic procedures and had a benign presentation and clinical course, the cause in this case is presumed to be related to copious retching during a bout of travel-associated gastroenteritis.

## Conclusions

Patients presenting to the ED with abdominal pain and severe retching are likely to undergo CT imaging to exclude emergent causes. Gastric pneumatosis can readily be identified on CT. Emergency physicians will likely have a high concern for life-threatening situations that lead to gastrointestinal pneumatosis. However, this case highlights a more benign case, likely caused by severe retching from travel-associated gastroenteritis. In the absence of sepsis, shock, lactic acidosis, profound comorbidities, or other evidence of critical illness, clinicians might consider benign causes of gastric pneumatosis identified on CT.
